# Isolation and Antimicrobial Activity of Coumarin Derivatives from Fruits of *Peucedanum luxurians* Tamamsch

**DOI:** 10.3390/molecules23051222

**Published:** 2018-05-20

**Authors:** Jarosław Widelski, Simon Vlad Luca, Adrianna Skiba, Ioanna Chinou, Laurence Marcourt, Jean-Luc Wolfender, Krystyna Skalicka-Wozniak

**Affiliations:** 1 Department of Pharmacognosy with Medicinal Plant Unit, Medical University of Lublin, Chodzki, 120-093 Lublin, Poland; jwidelski@pharmacognosy.org (J.W.); simon-vlad.v.luca@d.umfiasi.ro (S.V.L.); skiba.adrianna@gmail.com (A.S.); 2 Department of Pharmacognosy, “Grigore T. Popa” University of Medicine and Pharmacy, 16 Universitatii Street, 700115 Iasi, Romania; 3 Department of Pharmacognosy and Chemistry of Natural Products, School of Pharmacy, University of Athens, Zografou, 15771 Athens, Greece; ichinou@pharm.uoa.gr; 4 School of Pharmaceutical Sciences, EPGL, University of Geneva, University of Lausanne, CMU, 1, Rue Michel Servet, 1211 Geneva 4, Switzerland; Laurence.Marcourt@unige.ch (L.M.); Jean-Luc.Wolfender@unige.ch (J.-L.W.)

**Keywords:** *Peucedanum luxurians* Tamamsch., Apiaceae, accelerated solvent extraction, purification, natural products, countercurrent chromatography

## Abstract

As a continuation of searching for phytoconstituents that act as promising agents for antimicrobial therapy, rare coumarins were isolated from fruits of *Peucedanum luxurians* and tested. In a first step, the content of major compounds in the aerial parts and fruits of *P. luxurians* were compared. The results clearly showed that the fruits with dichloromethane as a solvent yielded, in most cases, higher concentrations of almost all the analyzed coumarins than the aerial parts, with peucedanin detected as the most abundant compound with a concentration of 4563.94 ± 3.35 mg/100 g. Under this perspective, the dichloromethane extract from the fruits of *P. luxurians* was further submitted to high performance countercurrent chromatography with a mixture of *n*-hexane, ethyl acetate, methanol, and water 6:5:6:5 (*v*/*v*). Combination of HPCCC and prep-HPLC yielded 6′,7′-dihydroxybergamottin (**1**), officinalin (**2**), stenocarpin isobutyrate (**3**), officinalin isobutyrate (**4**), 8-methoxypeucedanin (**5**), and peucedanin (**6**). Isolated compounds were tested against several Gram-positive and Gram-negative bacteria strains. 6′,7′-Dihydroxybergamottin, peucedanin, and officinalin isobutyrate appeared to be the most active against all tested bacteria strains with minimum inhibitory concentration (MIC) values between 1.20 and 4.80 mg/mL. To the best of our knowledge, this is the first report about countercurrent isolation of mentioned coumarins, as well as the first information about their antimicrobial activity.

## 1. Introduction

As stated in the WHO report, in the last few decades, the incidence of microbial infections has increased dramatically together with emergence of antimicrobial resistant strains [1]. Discovering some alternatives that can potentially be effective in the treatment of these infectious diseases is a major challenging issue worldwide and phytoconstituents are considered as promising agents for antimicrobial therapy [1].

Plants have been well-known sources of inspiration for developing novel drug compounds since antiquity [1]. Coumarins, naturally plant-derived compounds with benzopyrone moiety, possess a wide variety of biological activities. Series of coumarin analogues are being extensively studied due to their broad spectrum, low toxicity, and lower drug resistance properties [2]. Novobiocin, an aminocoumarin antibiotic produced by the actinomycete *Streptomyces niveus*, was launched onto the market in the mid-1950s as an effective antistaphylococcal agent used in the treatment of MRSA [3]. Different naturally-isolated coumarins, as well as their chemically modified analogs, are active against numerous bacterial strains, including those which developed multidrug resistance [2].

The genus *Peucedanum*, comprising between 100 and 120 species, and widely distributed across the world, is an important group of the Apiaceae family. Plants belonging to *Peucedanum* have been widely used to treat various diseases, including sore throat, coughs, colds, headaches [4,5], epilepsy, rheumatism, and cardiovascular and gastrointestinal problems [6,7]. The first information about the pharmacological application of *Peucedanum* plants can be found in “*The Canon of Medicine*”—an encyclopedia of medicine compiled by Persian philosopher Avicenna in 1025 [7]. In Central European medicine, the fruits of *P. alsaticum* and *P. cervaria* were found to be applied as expectorant, diuretic, diaphoretic, sedative, stomachic, and antimicrobial agents [8]. In Austrian traditional medicine, rhizomes of *P. ostruthium* have a very long history of use as a remedy for anti-inflammatory ailments [9]. Plants of the *Peucedanum* genus have been used for centuries as antibacterial agents and for some of them, the activity was confirmed by biological and pharmacological studies, showing moderate or high activity against different human pathogens. Such activities have also been reported for *P. paniculatum* (leaves and roots), *P. alsaticum, P. cervaria, P. graveolens, P. ruthenicum, P. zenkeri* fruits, or *P. ostruthium* roots [7]. Dichloromethane extracts of *P. ostruthium* roots have demonstrated anti-mycobacterial activity against *Mycobacterium fortuinum,* responsible for respiratory system infections. Two isolated furanocoumarins, ostruthin and isoimperatorin, were found to be the most potent, with low minimum inhibitory concentration (MIC) values similar to those of ethambutol and isoniazid [10]. Oxypeucedanin hydrate, isolated also from rhizomes of *P. ostruthium*, showed prominent activity against *Bacillus cereus* [11]*.* Imperatorin, bergapten, and isopimpinellin were responsible for the antimicrobial activity of methanolic extract of *P. zenkeri* seeds [12].

Due to the versatile and numerous therapeutic traditional uses, a number of phytochemical analyses were performed on *Peucedanum* representatives. Comparative phytochemical data are available for 50 species belonging to genus *Peucedanum* [7]. Among all the chemical constituents identified in the plants from *Peucedanum* genus, the highest impact on pharmacological activity is assigned to coumarins and essential oils (usually containing coumarin compounds) [7,13]. Coumarins with different molecular scaffolds have been isolated and identified in different *Peucedaum* such as: Simple coumarins and their glycosides; linear and angular furano- and dihydrofuranocoumarins and their glycosides; and linear and angular dihydropyranocoumarins and their glycosides [7].

*Peucedanum luxurians* Tamamsch. was found as an endemic plant from Armenia, growing in the area around Mount Ararat. Preliminary studies showed the presence of rare furanocoumarins with unique structures in the herb and fruits of this species [14]. For some of them, the immunomodulating activity was evaluated [15]. The aim of our study is to elaborate the efficient and fast procedure for their isolation by the use of high-performance countercurrent chromatography, as well as to evaluate their antimicrobial activity.

## 2. Results and Discussion

### 2.1. Preliminary HPLC Analysis and Screening of Activity of Extracts

Previously, few coumarins have been isolated from aerial parts of the *P. luxurians,* such as stenocarpin; stenocarpin isobutyrate; officinalin; officinalin isobutyrate; and 8-methoxypeucedanin together with xanthotoxin, isoimperatorin, bergaptene, peucedanin, and cnidilin [14] where few steps of column chromatography were applied. Because the fruits of Apiaceae plants are usually the best source of these compounds, in the first step of the experiment, the major compounds in the aerial parts and fruits of *P. luxurians* were compared. Additionally, the influence of different solvents on extraction efficiency was investigated. The procedure for the extraction (accelerated solvent extraction) and all the other parameters (temperature, time, amount of plant material) were the same for each sample.

**Figure 1:**
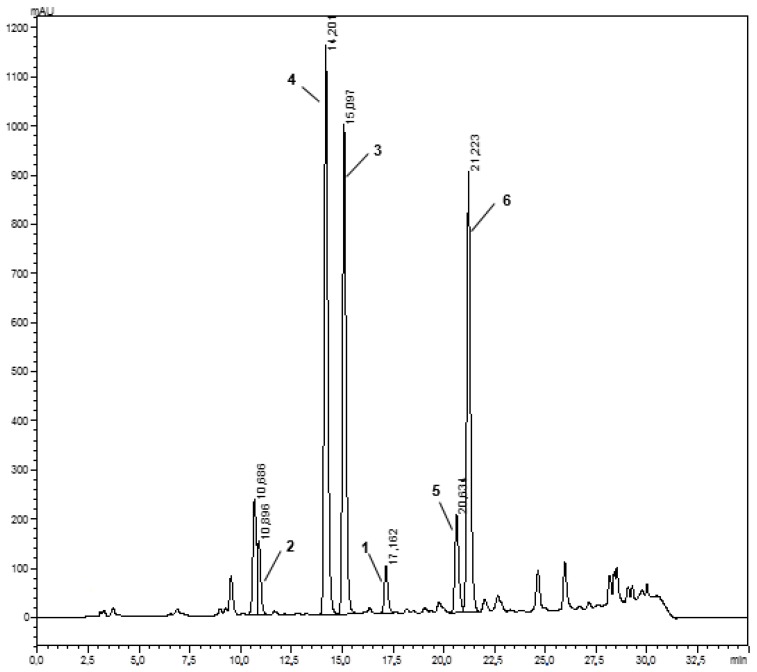
HPLC-DAD chromatogram of crude dichloromethane extract from fruits of *P. luxurians*.

Obtained extracts prepared for quantitative analysis were injected to HPLC with diode-array detection (DAD) and in the first step, the area under each peak was compared. Additionally, tentative qualitative analysis was performed using an LC–MS system. Six coumarin derivatives were identified and their presence was confirmed in each analyzed extract. The results clearly showed that the fruits yielded higher concentrations of almost all analyzed coumarins compared to the aerial parts and dichloromethane produced the highest yield for almost all coumarins. At the same time, dichloromethane and methanolic extracts from both the fruits and aerial parts were subjected to preliminary screening for their antibacterial activity, which showed that those from fruits were more active against most of the test pathogens. Under this perspective, the dichloromethane extract of *P. luxurians* fruits was further submitted to high-performance counter-current chromatography (HPCCC) separation to isolate pure compounds for evaluation of their biological activity, as well as to complete the quantitative analyses (HPLC-DAD chromatogram of the crude extract is presented in Figure 1).

### 2.2. HPCCC Separation

In order to achieve an efficient separation, different two-phase solvent systems were tested. Mixtures of *n*-hexane, ethyl acetate, methanol, and water (HEMWat) were proven to be very efficacious in the separation of coumarins, including coumarins from other species of *Peucedanum* genus [16]. The K value, which is a partition coefficient for the target compounds, were calculated by dividing the concentration of the analyte in the upper stationary phase by that in the lower mobile phase (reversed mode) [16]. Among the three HEMWat solvent mixtures tested, only the one with a ratio of 6:5:6:5 (*v*/*v*/*v*/*v*) gave the most appropriate K values of the analyzed compounds (from 0.19 to 3.61; Table 1). Furthermore, the separation factor (*α =* K_2_/K_1_, K_2_ > K_1_) was found to be optimal (*α* > 1.20) for all target compounds, except compounds **3** and **4** (see Table 1), suggesting these two compounds may co-elute under HPCCC experimental conditions. In this regard, two other solvent systems, one consisting of petroleum ether, ethyl acetate, methanol, and water (1:1:1:1 *v*/*v*/*v*/*v*) and the other of *n*-hexane, acetone, and water (3.9:5:1.9 *v*/*v*/*v*) were investigated. Despite the fact that the K values of compounds **3** and **4** were adequate, the separation factor *α* still remained unsolved in both situations. Nevertheless, the K values of some of the compounds of interest were either lower than 0.2 (which would give a low peak resolution with elution near the solvent front), or higher than **5** (which would lead to an excessive broadening of peak with a longer elution time). Thus, HEMWat 6:5:6:5 (*v*/*v*/*v*/*v*) was further selected as HPCCC separation solvent system. With this solvent system, at 1600 rpm and a flow rate of 6 mL/min in the semi-preparative separation mode, the retention of the stationary phase was stable and was equal to 78%.

Following a single HPCCC injection of the dichloromethane extract of *P. luxurians* fruits (300 mg), five fractions were collected: FR I (min 15–16), FR II (min 25–26), FR III (min 28–30), FR IV (min 44–46) and FR V (min 54–59) (Figure S1). All five fractions were collected within 60 min, evaporated, and dissolved in methanol prior to HPLC-DAD analysis. FR I, II, and IV contained compounds **1**, **2**, and **5**, respectively, with purities higher than 80% but lower than 95%. A mixture of compounds **3** and **4** was found in FR III, whereas FR V gave compound **6** with 99.7% purity. In order to obtain higher amounts of the target compounds, separation was repeated two times more (each time with 300 mg of extract) and all similar fractions were pooled together with the previously collected ones, to yield 10 mg of FR I, 5 mg of FR II, 15 mg of FR III, 5 mg of FR IV, and 68 mg of FR V (compound **6**, 99.7% purity).

**Figure 2:**
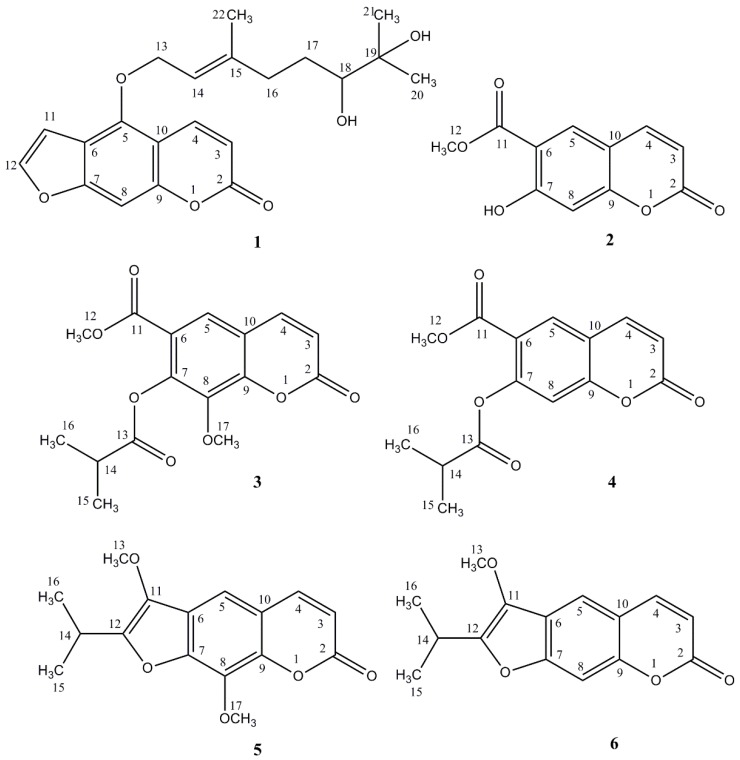
Chemical structures of coumarins isolated from *P. luxurians*: 6′,7′-dihydroxybergamottin (**1**), officinalin (**2**), stenocarpin isobutyrate (**3**), officinalin isobutyrate (**4**), 8-methoxypeucedanin (**5**), and peucedanin (**6**).

Fractions I-IV were subjected to semi-preparative HPLC separation, separately. Five compounds were obtained with purity above 97% (as measured by HPLC-DAD): Compound **1** (6 mg, 98.4% purity), compound **2** (2 mg, 97.1% purity), compound **3** (6 mg, 99.7% purity), compound **4** (7 mg, 97.8% purity), and compound **5** (1.5 mg, 99.7% purity). The structures of compounds were elucidated by complementary spectroscopic methods, such as: HPLC-DAD connected to electrospray-ionisation quadrupole time-of-flight mass spectrometer (HPLC-DAD-ESI-Q-TOF-MS) and 1D- and 2D-NMR (Supplementary Material). The compounds were determined as 6′,7′-dihydroxybergamottin (**1**), officinalin (**2**), stenocarpin isobutyrate (**3**), officinalin isobutyrate (**4**), 8-methoxypeucedanin (**5**), and peucedanin (**6**). These data were in agreement with those available in the literature [14,17,18]. Their chemical structures are presented in Figure 2.

Except for 6′,7′-dihydroxybergamottin (**1**), which is a well-known cytochrome P450 3A4 inhibitor from grapefruit juice that was not reported in *Peucedanum* genus [17], all the other coumarins were identified in the aerial parts of *P. luxurians* [14,15]. Officinalin (**2**), officinalin isobutyrate (**4**), and peucedanin (**6**) were also isolated from the dichloromethane fraction of the essential oil of *P. tauricum* Bieb. fruits [18], while 8-methoxypeucedanin (**5**) was previously reported in the chloroform extract of *P. ruthenicum* M.B roots [19] and the hexane-diethyl ether-methanol extract of *P. longifolium* Waldst. and Kit. roots [20]. Peucedanin (**6**) and officinalin isobutyrate (**4**) were isolated also from the etheric extract of the roots of *Opopanax chironium* (L.) Koch. (Apiaceae) [21], while peucedanin (**6**) and officinalin (**2**) were isolated from the chloroform extract of the aerial parts of *Opopanax hispidus* (Friv.) Griseb [22]. Moreover peucedanin (**6**) was purified from the petroleum ether-diethyl ether extract of *P. officinale* L. fruits [20] and the diethyl ether, methyl-*tert*-butyl ether, and ethyl acetate extracts of *P. morisonii* Bess. aerial parts [23]. In addition, stenocarpin isobutyrate (**3**) was isolated from the methanol and ethyl acetate extracts of *P. morisonii* Bess., aerial parts [23]. Although these coumarins were identified in *Peucedanum* genus (except 6′,7′-dihydroxybergamottin), their isolation through HPCCC technique was not previously reported.

### 2.3. Quantitative HPLC-DAD Analysis

The purified compounds were further used to complete quantitative analysis, as well to evaluate their potential antibacterial activity. Parameters of the calibration curves and both the limit of detection (LOD) and limit of quantification (LOQ) values are presented in Table S1 (Supplementary Materials). Relative standard deviation (RSD%), which is a measure of precision, was lower than 6.05% in fruit extracts and 5.97% in aerial part extracts (Table 2). Selection of the appropriate extraction methods is an important issue in phytochemistry. An appropriately selected method should ensure the maximum recovery of the requisite active substances in the shortest possible time and at the lowest financial cost. As pressurized liquid extraction (accelerated solvent extraction; ASE) was previously shown to give the highest yield of simple coumarins and furanocoumarins from plants from Apiaceae family [24,25], this technique was used to elaborate the optimal conditions for extraction of target compounds. Due to the better penetration of the sample by the solvent, ASE ensures complete extraction in short time with a small volume of organic solvent [25]. A temperature of 100 °C and 10 min static time were selected for comparison of extraction efficiency in currently presented experiments. The results clearly showed that fruits yielded higher concentrations of all analyzed coumarins compared to those observed in the aerial parts of *P. luxurians*. With respect to the extraction solvent, dichloromethane gave, in most cases, a more efficient extraction of the compounds of interest from the fruits as compared to methanol and petroleum ether (Table 2). Peucedanin (**6**) was detected as the most abundant compound in the crude dichloromethane extract with a concentration of 4563.94 ± 3.35 mg/100 g.

### 2.4. Antimicrobial Activity

The isolated compounds were subjected to further antimicrobial evaluation and results were compared to reference drug, netilmicin. All coumarins revealed broad diversity regarding growth-inhibitory activity (Table 3) with 6′,7′-dihydroxybergamottin (**1**) as the most active against all tested bacteria strains (inhibition zone between 16 to 17 mm and MIC values between 1.20 and 2.10 mg/mL). Similar activity was noticed for peucedanin (**6**) and officinalin isobutyrate (**4**) (inhibition zone between 16 to 13 mm and MIC values between 1.40 and 4.80 mg/mL), while the rest of the tested coumarins showed moderate activity (inhibition zone between 12 to 14 mm and MIC values between 3.90 and 5.75 mg/mL).

Officinalin isobutyrate (**4**) was more active than officinalin (**2**) itself (MIC values 2.25–4.80 and 4.50–5.75 mg/mL, respectively), except for the inhibition *E. coli* growth, where both compounds had almost same activity. According to previously published data, the stronger activity was a result of the aliphatic chain substituted in C-7 position, e.g., an isoprenyl group to the furanocoumarin skeleton resulted in an increase in lipophilicity of the molecule, what helped to penetrate through the thick membrane of these bacteria [10,26]. This aliphatic chain substituted at C-5 position shared responsibility for activity of 6′,7′-dihydroxybergamottin (**1**) which was the most active in our experiments. When a methoxy group was attached to C-8 position in stenocarpin isobutyrate (**3**), the compound exhibited a weak antibacterial activity, as compared to its structural analogue, officinalin isobutyrate (**4**). Similarly, the presence of methoxy group in the ring of 8-methoxypeucedanin (**5**) led to a decrease in activity when compared to peucedanin (**6**). However previously published reports suggest that additional methoxygroup in the C-8 position for another furanocoumarin-phellopterin may enhance the antimicrobial activity [26]; thus, further experiments are required. To the best of our knowledge, this is the first report concerning the antimicrobial activity of isolated compounds.

## 3. Materials and Methods

### 3.1. Chemicals

Analytical-grade petroleum ether, *n*-hexane, dichloromethane, ethyl acetate, acetone, and methanol were purchased from POCh (Gliwice, Poland). Methanol, acetonitrile, ammonium formate, and formic acid used for HPLC and LC-MS analyses were of chromatographic grade and obtained from J.T. Baker (Deventer, The Netherlands). Deuterated chloroform (CDCl_3_, 99.8 atom %D) was acquired from Armar Chemicals (Döttingen, Switzerland). A Simplicity^®^ water purification system (Millipore, France) was used to purify the water.

### 3.2. Plant Material

The plant material (mature fruits and herb of *Peucedanum luxurians* Tamamsch.) used in the experiments was obtained in 2016 from the Botanical Garden of Adam Mickiewicz University in Poznań, Poland (plant material identified by Grażyna Naser). The voucher specimen (7973_S003) was kept in the Department of Pharmacognosy, Medical University of Lublin, Poland. The air-dried fruits and herbs were dried, grounded and pulverized.

### 3.3. Accelerated Solvent Extraction

A Dionex ASE 100 instrument (Dionex, Sunnyvale, CA, USA) was used for solvent extraction. Exactly 1 g of dried plant material was placed into a 10-mL stainless-steel extraction cell. In order to optimize the extraction parameters, the aerial parts and fruits were extracted with three different solvents (petroleum ether, dichloromethane and methanol) at 100 °C for a static time of extraction of 10 min in one cycle. The extracts that resulted were evaporated to dryness, dissolved in methanol in 25-mL calibration flasks, and subjected to quantitative HPLC-DAD analysis.

### 3.4. HPCCC Separation

#### 3.4.1. HPCCC Apparatus

A spectrum high-performance counter-current chromatograph (HPCCC) instrument (Dynamic Extractions Ltd., Slough, Berkshire, UK) equipped with two multilayer polytetrafluorethylene coils, one for analytical (22 mL) and the other for semi-preparative purpose (136 mL), was used. The temperature was maintained at 30 °C and maximum revolution speed (1600 rpm) was set up to obtain the maximum centrifugal force (240× *g*). The HPCCC system was connected to an Alpha 10 pump and a Sapphire UV detector (ECOM, Prague, Czech Republic).

#### 3.4.2. Selection of Two-Phase Solvent System

In order to calculate the partition coefficients (K) of the target compounds, approx. 1 mg of crude extract was mixed in the test tubes with 2 mL of each phase of selected solvent system. After shaking, it was left to stand at room temperature until complete separation of the layers. Equal volumes (0.5 mL) of the upper and lower phases were taken, evaporated to dryness, redissolved in methanol (1 mL), and analyzed by HPLC-DAD.

#### 3.4.3. Separation Procedure

The HEMWat mixture selected for HPCCC separation was equilibrated at room temperature in a separatory funnel and the two phases were separated before analysis and degassed for 10 min. The multilayer semi-preparative coil was firstly filled with the stationary phase (upper). The rotation of the column was set to 1600 rpm, then the mobile phase (lower) was pumped into the column at a flow rate of 6 mL/min. After the hydrodynamic equilibrium was attained (as indicated by the absence of the stationary phase flowing from the column), the sample solution, containing 300 mg of the crude dichloromethane extract of *P. luxurians* fruits dissolved in 3 mL of each phase of the selected solvent system, was injected. The effluent from the column was monitored at 254 nm and the collected fractions were evaporated and dissolved in methanol (1 mL) before HPLC-DAD analysis. The HPCCC separation was repeated two more times and the fractions containing target compounds were pooled together and evaporated to dryness.

### 3.5. Semi-Preparative HPLC Separation

Four fractions obtained after HPCCC separation (FR I–IV) were dissolved in methanol (10 mg/mL) and further purified by semi-preparative HPLC, which was carried out on a Hitachi LaChrom 7000 HPLC system (Hitachi Ltd., Tokyo, Japan) equipped with a D-7000 interface, an L-7150 pump, an L-7420 DAD detector, and an Advantec SF-3120 fraction collector. Separation was conducted on a Cosmosil C18-AR-II (250 mm × 10 mm, 5 μm) by injecting 50 μL of sample; the mobile phase consisted of 60% methanol for fraction FR I, 50% methanol for fractions FR II and FR III and 65% methanol for fraction FR IV; the flow rate was 4 mL/min and the effluent was monitored at 254 nm.

### 3.6. HPLC-DAD Analysis

Analytical HPLC-DAD analysis was performed using Shimadzu HPLC equipment (Shimadzu, Tokyo, Japan) coupled with an automatic degasser (DGU-20A 3R), a quaternary pump (LC-20AD), an auto-sampler (SIL-20A HT), a DAD detector (SPD-M20A), and an Agilent Zorbax Eclipse XDB-C18 (250 mm × 4.6 mm, 5 µm) column. Water (A) and methanol (B) with the following gradient was used: 0 min, 50% B; 5 min, 60% B; 25 min, 80% B; and 30–35 min 100% B. The flow rate was 1 mL/min; the column temperature was 25°C, the injection volume was 10 μL and the detection wavelength was set at 254 nm.

All purified compounds, identified as 6′,7′-dihydroxybergamottin (**1**), officinalin (**2**), stenocarpin isobutyrate (**3**), officinalin isobutyrate (**4**), 8-methoxypeucedanin (**5**), and peucedanin (**6**), were dissolved in methanol (0.1 mg/mL) and the stock solutions were diluted in a series of appropriate concentrations (20–100 μg/mL) for the construction of calibration curves. The results of the quantitative analysis were expressed as mean ± standard deviation mg compound/100 g of dry weight plant material. The LOD and LOQ values were calculated from the standard deviation of y-intercepts (*σ*) and the slopes (*S*), according to the following formulas: LOD = 3.3 × *σ/S* and LOQ = 10 × *σ/S*. All experiments were repeated three times.

### 3.7. HPLC-DAD-ESI-Q-TOF-MS Analysis

HPLC-DAD-ESI-Q-TOF-MS experiments were performed on an Agilent 1200 HPLC (Agilent Technologies, Santa Clara, CA, USA) coupled to an auto-sampler (G1329B), a binary pump (G1312C), a column oven (G1316A), and a DAD detector (G1315D). The Agilent G6530B ESI-Q-TOF-MS was equipped with a nitrogen generator, a compressed air generator, and a compressed air cylinder. Separation was carried out on a Phenomenex Gemini C18 (100 × 2 mm, 3 µm) column with 60% acetonitrile, 10 mM ammonium formate, and 0.1% formic acid in water (solvent A) and 95% acetonitrile, 10 mM ammonium formate, and 0.1% formic acid in water (solvent B); under the following conditions: 0−10 min, 40% B; 30 min, 80% B; and 35 min, 90% B. The total analysis time was 35 min, post-time was 12 min, injection volume was 10 μL, flow was 0.2 mL/min, and DAD spectra were recorded from 200 to 400 nm. ESI-Q-TOF-MS analysis was performed in positive mode, as follows: mass range 100–1000 *m*/*z*, gas temperature 350 °C, nitrogen flow 10 L/min, nebulizer pressure 40 psig, skimmer 65 V, capillary voltage 4000 V, fragmentor 120 V, and collision energies 10 and 40 eV.

### 3.8. NMR Analysis

A Bruker Avance III HD 600 MHz NMR spectrometer equipped with a QCI 5 mm Cryoprobe and a SampleJet automated sample changer (Bruker BioSpin, Rheinstetten, Germany) was employed for 1D-NMR (^1^H and ^13^C-NMR in CDCl_3_) and 2D-NMR (correlation spectroscopy, COSY; heteronuclear multiple-bond correlation, HMBC; heteronuclear single-quantum correlation, and HSQC) spectroscopy. Chemical shifts are reported in parts per million (δ) using the residual CDCl_3_ signal (δ_H_ 7.26; δ_C_ 77.2) as internal standards for ^1^H and ^13^C NMR, respectively; coupling constants (*J*) are given in Hz.

### 3.9. Antimicrobial Activity

The antimicrobial activities of extracts and isolated coumarins were determined using the diffusion and dilution techniques and by measuring their MICs against two Gram-positive bacteria, *Staphyloccocus aureus* (ATCC 25923) and *Staphylococcus epidermidis* (ATCC 12228), as well as four Gram-negative *Pseudomonas aeruginosa* (ATCC 27853), *Escherichia coli* (ATCC 25922), *Enterobacter cloacae* (ATCC 13047), and *Klebsiella pneumoniae* (ATCC 13883), according to a previously published method [27].

The extracts and test compounds were dissolved before the assay in methanol (5 mg/mL). For each experiment, control disks with pure solvent were used as a blind control. Petri dishes had been previously inoculated with the tested microorganisms to give a final cell concentration of 10^7^ cells/mL. Ten µL volumes of the above solutions were required to wet the test paper discs. The incubation conditions used in experiments were 24 h at a temperature of 37 °C. Standard antibiotic netilmicin (4–88 μg/mL) was used as reference drug. For each experiment, any pure solvent used was also applied as a blind control. The results were reported as the diameter of the zone of inhibition around each disk (in mm) and MIC values were calculated. The MIC values were determined using the dilution method in 96-well plates. The experiments were repeated three times and the results were expressed as average values. Netilmicin was used as reference compound.

## 4. Conclusions

An efficient strategy based on elaboration of optimal extraction parameters as well as one step HPCCC for the rapid separation, and purification of rare coumarins from the nonpolar extract of *P. luxurians*, was presented. This was the first report concerning the efficient isolation of coumarin derivatives such as: 6′,7′-dihydroxybergamottin, officinalin, stenocarpin isobutyrate, officinalin isobutyrate, 8-methoxypeucedanin, and peucedanin with HPCCC. Additionally, 6′,7′-dihydroxybergamottin was reported for the first time in *Peucedanum* genus. The isolated compounds were evaluated for their antibacterial activity for the first time and possible structure–activity relation was discussed.

## Figures and Tables

**Table 1:** Partition coefficients (K values) of coumarins from *P. luxurians.*

Solvent System	Coumarins					
	1	2	3	4	5	6
HEMWat (5:6:5:6)	0.83	3.01	5.14	5.16	9.49	11.57
HEMWat (1:1:1:1)	0.47	1.59	3.09	3.25	5.31	6.34
PEMWat (1:1:1:1)	0.50	1.95	2.47	2.51	4.38	4.90
HAtWat (3.9:5:1.9)	0.16	1.22	1.15	1.06	0.55	0.33
**HEMWat (6:5:6:5)**	**0.19**	**0.89**	**1.57**	**1.65**	**2.85**	**3.61**
	*α*_2/1_ = 4.68	*α*_3/2_ = 1.76	*α*_4/3_ = 1.05	*α*_5/4_ = 1.73	*α*_6/5_ = 1.27	

HEMWat denotes hexane-ethyl acetate-methanol-water, PEMWat denotes petroleum ether-ethyl acetate-methanol-water, HAtWat denotes hexane-acetone-water, and α denotes the separation factor.

**Table 2:** Quantitative analysis results of coumarins detected in different extracts of *P. luxurians*.

Coumarins	Plant Material	Solvent	Yield (mg/100 g d.w.)	RSD%
**1**	Aerial parts	PE	13.72 ± 0.41	2.99
		DCM	24.71 ± 0.66	2.67
		MeOH	30.61 ± 1.83	5.97
	Fruits	PE	83.17 ± 5.04	6.05
		DCM	442.23 ± 2.45	0.55
		MeOH	465.74 ± 1.66	0.36
**2**	Aerial parts	PE	29.60 ± 0.84	2.84
		DCM	51.70 ± 2.71	5.24
		MeOH	53.51 ± 0.39	0.73
	Fruits	PE	229.12 ± 0.62	0.27
		DCM	1336.26 ± 0.80	0.06
		MeOH	1309.43 ± 1.31	0.10
**3**	Aerial parts	PE	23.61 ± 0.02	0.09
		DCM	33.49 ± 0.19	0.58
		MeOH	47.84 ± 0.95	1.99
	Fruits	PE	56.86 ± 0.03	0.05
		DCM	101.72 ± 0.10	0.10
		MeOH	93.88 ± 0.59	0.63
**4**	Aerial parts	PE	8.39 ± 0.12	0.13
		DCM	8.56 ± 0.16	0.12
		MeOH	8.47 ± 0.41	1.06
	Fruits	PE	89.93 ± 0.12	0.13
		DCM	191.78 ± 0.02	0.01
		MeOH	170.06 ± 0.09	0.05
**5**	Aerial parts	PE	62.55 ± 0.03	0.05
		DCM	79.68 ± 0.29	0.37
		MeOH	66.21 ± 0.07	0.11
	Fruits	PE	341.91 ± 0.17	0.05
		DCM	1652.15 ± 0.87	0.05
		MeOH	1622.91 ± 0.56	0.03
**6**	Aerial parts	PE	28.94 ± 0.11	0.38
		DCM	22.60 ± 0.11	0.49
		MeOH	21.49 ± 0.25	1.16
	Fruits	PE	3689.91 ± 1.07	0.03
		DCM	4563.94 ± 3.35	0.07
		MeOH	4538.09 ± 1.13	0.02

PE—petroleum ether; DSM—dichloromethane; MeOH—methanol.

**Table 3:** Activity of *P. luxurians* extracts and isolated coumarins against tested bacteria (zone of inhibition (mm)/MIC values (mg/mL)).

Tested Extract/Compound	*S. aureus*	*S. epidermidis*	*P. aeruginosa*	*E. cloacae*	*K. pneumoniae*	*E. coli*
*P. luxurians* aerial parts DCM	15/1.90	16/1.88	13/2.40	12/3.50	12/3.10	12/3.35
*P. luxurians* aerial parts MeOH	17/0.90	17/0.92	13/2.80	12/3.50	12/2.75	12/2.50
*P. luxurians* fruits DCM	17/0.84	17/0.90	14/3.00	13/3.45	13/2.77	13/2.60
*P. luxurians* fruits MeOH	18/0.95	18/0.85	14/2.84	14/2.75	14/2.50	14/2.25
(1) 6′,7′-Dihydroxybergamottin	17/1.20	17/1.35	17/1.37	16/1.75	16/2.10	17/1.45
(2) Officinalin	13/4.50	12/5.50	12/5.00	12/5.75	13/4.80	13/4.90
(3) Stenocarpin isobutyrate	12/5.25	14/4.00	13/5.00	13/4.80	14/3.90	14/4.50
(4) Officinalin isobutyrate	14/3.50	15/2.70	14/3.50	15/2.75	15/2.25	13/4.80
(5) 8-metoxypeucedanin	12/5.25	14/4.00	13/5.00	13/4.80	14/3.90	14/4.50
(6) Peucedanin	16/1.50	16/1.75	17/1.40	16/2.10	16/2.50	16/2.75
Netilmicin	21/0.004	25/0.004	20/0.088	23/0.008	22/0.008	24/0.010

## Supplementary Materials

The following are available online. Figure S1: HPCCC chromatogram of the dichloromethane extract of *Peucedanum luxurians* fruit, Figure S2: HPLC-DAD chromatograms and UV spectra of isolated compounds, Table S1: Parameters of calibration curves of quantitative HPLC-DAD analysis, and Spectroscopic data of compounds 1–6.
