# Supplementary material for: Isolation and Antimicrobial Activity of Coumarin Derivatives from Fruits of Peucedanum luxurians Tamamsch
Source: Molecules. 2018 May 20;23(5):1222. doi: 10.3390/molecules23051222 (PMC6100078; doi:10.3390/molecules23051222)
Supplement: Supplementary file 1 [file molecules-23-01222-s001.pdf]

Supplementary Material for

Isolation and antimicrobial activity of coumarin derivatives from fruits of *Peucedanum luxurians* Tamamsch.

Jarosław Widelski [1], Simon Vlad Luca[1,2], Adrianna Skiba[1], Ioanna Chinou [3], Laurence Marcourt [4], Jean-Luc Wolfender [4], Krystyna Skalicka-Wozniak [1,*]

[1]*Department of Pharmacognosy with Medicinal Plant Unit, Medical University of Lublin, Chodzki 1, 20-093 Lublin, Poland (kskalicka@pharmacognosy.org)*

[2]*Department of Pharmacognosy, "Grigore T. Popa" University of Medicine and Pharmacy, 16 Universitatii Street, 700115 Iasi, Romania*

[3]*Department of Pharmacognosy and Chemistry of Natural Products, School of Pharmacy, University of Athens, Zografou, 15771 Athens, Greece*

[4]*School of Pharmaceutical Sciences, EPGL, University of Geneva, University of Lausanne, CMU, 1, Rue Michel Servet, 1211 Geneva 4, Switzerland*

Correspondence:

Assoc. Prof. Krystyna Skalicka-Woźniak, Department of Pharmacognosy with Medicinal Plant Unit, Medical University of Lublin, 1 Chodzki Str., 20-093 Lublin, Poland, E-mail address: kskalicka@pharmacognosy.org Phone: +48814487086, fax: +48814487080

Part A. Figure S1. HPCCC chromatogram of the dichloromethane extract of *Peucedanum luxurians* fruit

Figure S2. Figure S2. HPLC-DAD chromatograms and UV spectra of isolated compounds

Part B. Table S1. Parameters of calibration curves of quantitative HPLC-DAD analysis

Part C. Spectroscopic data of isolated compounds

Part A

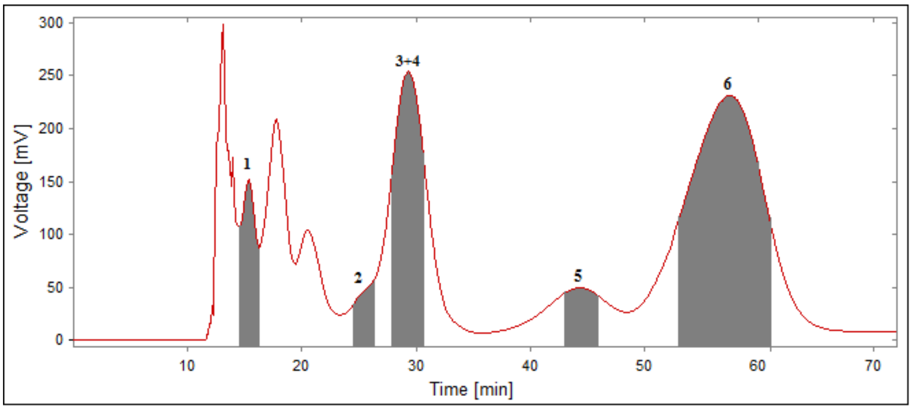

Figure S1. HPCCC chromatogram of the dichloromethane extract of *Peucedanum luxurians* fruits; solvent system: *n*-hexane-ethyl acetate-methanol-water (6:5:6:5, v/v/v/v); stationary phase: upper phase; mobile phase: lower phase; flow rate: 6 mL/min; revolution speed: 1600 rpm; stationary phase retention: 78%; detection: 254 nm; sample size: 300 mg of crude extract.

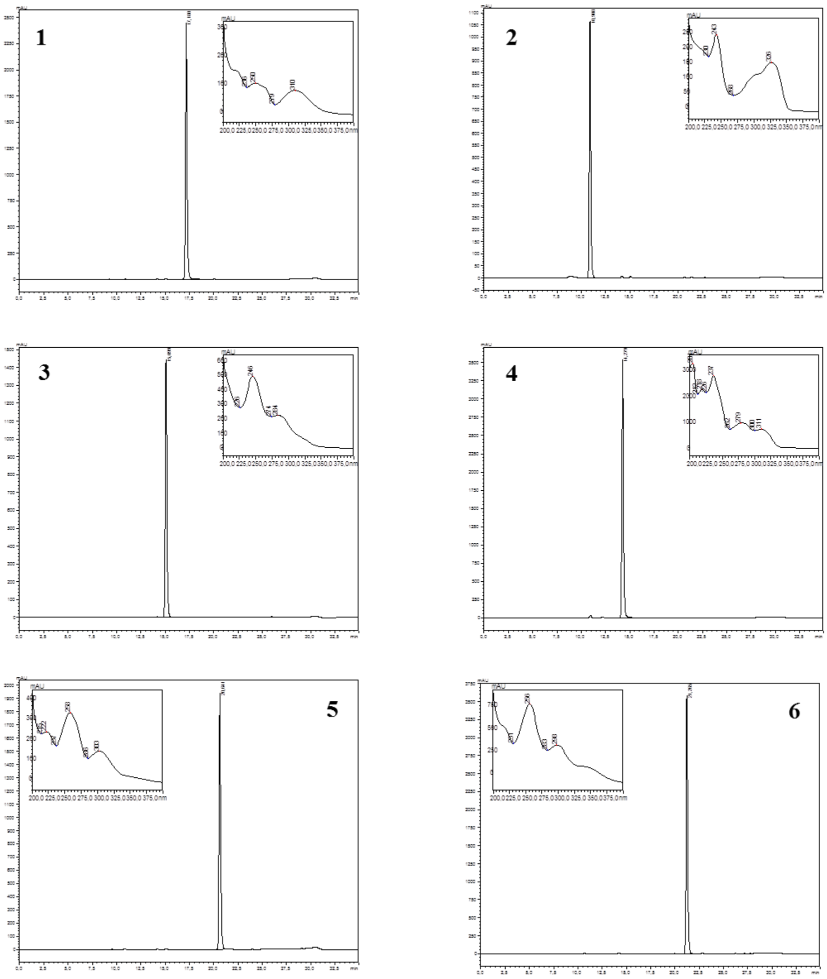

Figure S2. HPLC-DAD chromatograms and UV spectra of isolated compounds: (**1**) 6',7'-dihydroxybergamottin, (**2**) officinalin, (**3**) stenocarpin isobutyrate, (**4**) officinalin isobutyrate, (**5**) 8-methoxypeucedanin, (**6**) peucedanin

Formatted: Font color: Red

Part B.

Table S1. Parameters of calibration curves of quantitative HPLC-DAD analysis

Compound	Linear range (µg/mL)	Regression equation	R^2	LOD (µg/mL)	LOQ (µg/mL)
(**1**) 6',7'-Dihydroxybergamottin	20–100	y = 7235.8x – 8353.3	0.9998	0.86	2.62
(**2**) Officinalin	20–100	y = 6144x – 5744.9	0.9999	0.75	2.28
(**3**) Stenocarpin isobutyrate	20–100	y = 26320x – 16743	0.9990	0.63	1.91
(**4**) Officinalin isobutyrate	20–100	y = 40992x – 25363	0.9999	0.91	2.76
(**5**) 8-Methoxypeucedanin	20–100	y = 64647x – 64718	0.9999	0.73	2.23
(**6**) Peucedanin	20–100	y = 41911x - 29461	0.9999	0.74	2.26

LOD, limit of detection, LOQ, limit of quantification

Part C.

Spectroscopic data of isolated compounds

6',7'-Dihydroxybergamottin (**1**): $C_{21}H_{24}O_6$, MW 372.1573; UV (methanol, λ_{max}, nm): 225, 236 sh, 250, 279 sh, 310; ESI-MS: *m/z* 395.1455 $[M+Na]^+$ (calcd for $C_{21}H_{24}NaO_6$ 395.1465, Δ = 2.56 ppm); MS/MS (10 eV) *m/z* (rel. int.): 225.0154 (2), 193.1194 (3); ^{1}H NMR ($CDCl_3$, 600 MHz) δ 8.10 (1H, dd, *J*=9.7, 0.~~6~~ 8 Hz, H-4), 7.54 (1H, d, *J*=2.3 Hz, H-12), 7.10 (1H, t, *J*=0.8 Hz, H-8), 6.89 (1H, dd, *J*=2.3, ~~1.0~~0.8 Hz, H-11), 6.22 (1H, d, *J*=9.7 Hz, H-3), 5.54 (1H, tq, *J*=6.9, 1.3 Hz, H-14), 4.89 (2H, d, *J*=6.~~8~~ 9 Hz, H-13), 3.25 (1H, d, *J*=10.5 Hz, H-18), 2.30 (1H, ~~ddd, *J*=14.5, 9.7, 5.1 Hz~~m, H-16'), 2.09 (1H, m, H-16''), 1.64 (3H, d, *J*=1.3 Hz, H-22), 1.53 (1H, m, H-17'), 1.38 (~~2H~~1H, ~~dddd, *J*=13.9, 10.6, 9.4, 5.0 Hz~~m, H-17), 1.14 (3H, s, H-21), 1.10 (3H, s, H-20); ^{13}C NMR ($CDCl_3$, 151 MHz) δ 161.4 (C-2), 158.3 (C-7), 152.8 (C-9), 149.0 (C-5), 145.1 (C-12), 143.1 (C-15), 139.7 (C-4), 119.5 (C-14), 114.4 (C-6), 112.8 (C-3), 107.7 (C-10), 105.1 (C-11), 94.5 (C-8), 78.0 (C-18), 73.2 (C-19), 69.8 (C-13), 36.7 (C-16), 29.6 (C-17), 26.7 (C-21), 23.5 (C-20), 16.8 (C-22); in agreement with published data (Edwards et al., 1996; Tatum and Berry, 1979).

Officinalin (**2**): $C_{11}H_8O_5$ MW 220.0381; UV (methanol, λ_{max}, nm): 243, 268 sh, 326; ESI-MS: *m/z* 221.0454 $[M+H]^+$ (calcd for $C_{11}H_9O_5$ 221.0444, Δ = 4.32 ppm); MS/MS (40 V) *m/z* (rel. int.): 189.0081 (4), 161.0192 (3), 145.0286 (49), 133.0296 (35), 117.0340 (5), 105.0345 (52), 101.0345 (21), 89.0402 (59), 77.0404 (100), 63.0254 (50); ^{1}H NMR ($CDCl_3$, 600 MHz) δ 7.96 (1H, s, H-5), 7.55 (1H, d~~d~~, *J*=9.6~~, 0.7 Hz~~, H-4), 6.82 (1H, s, H-8), 6.22 (1H, d, *J*=9.6 Hz, H-3), 3.94 (3H, s, H-12); ^{13}C NMR ($CDCl_3$, 151 MHz) δ 169.6 (C-11), 164.4 (C-7), 160.2 (C-2), 159.1 (C-9), 143.1 (C-4), 130.8 (C-5), 114.3 (C-3), 112.1 (C-10), 110.2 (C-6), 105.0 (C-8), 52.9 (C-12); in agreement with published data (Tesso et al., 2005).

Stenocarpin isobutyrate (**3**): $C_{16}H_{16}O_7$ MW 320.0883; UV (methanol, λ_{max}, nm): 246, 274 sh, 284; ESI-MS: *m/z* 321.0956$[M+H]^+$ (calcd for $C_{16}H_{17}O_7$ 321.0969, Δ = 3.52 ppm); MS/MS (40 eV) *m/z*, (rel. int.): 219.09286 (100), 204.0013 (86), 191.0314 (8), 176.0108 (37), 159.0065 (69), 148.0132 (13), 131.0142 (15); MS/MS (10 eV) *m/z* (rel. int.): 251.0534 (100), 219.0283 (28); ^{1}H NMR ($CDCl_3$, 600 MHz) δ 7.84 (1H, s, H-5), 7.64 (1H, d, *J*=9.6 Hz, H-4), 6.39 (1H, d, *J*=9.6 Hz, H-3), 3.95 (3H, s, H-17), 3.82 (3H, s, H-12), 2.89 (1H, hept, *J*=7.0 Hz, H-14), 1.33 (6H, d, *J*=7.0 Hz, H-15, 16); ^{13}C NMR ($CDCl_3$, 151 MHz) δ 174.8 (C-13), 164.0 (C-11), 159.0 (C-2), 150.6 (C-9), 146.8 (C-7), 143.3 (C-4), 140.7 (C-8), 125.4 (C-5), 120.9 (C-6), 117.3 (C-10), 117.0 (C-3), 62.0 (C-17), 52.6 (C-12), 34.3 (C-14), 19.0 (C-15, 16); in agreement with published data (Chinou et al., 2007; Schults et al., 2003).

Officinalin isobutyrate (**4**): $C_{15}H_{14}O_6$ MW 290.078; UV (methanol, λ_{max}, nm): 237, 262 sh, 279, 300 sh, 311; ESI-MS: *m/z* 291.0863 $[M+H]^+$ (calcd for $C_{15}H_{15}O_6$ 291.0863, Δ = 3.15 ppm); MS/MS (10 eV): 221.0421 (100), 189.0196 (20); MS/MS (40 eV) *m/z* (rel. int.): 189.0164 (98), 161.0226 (24), 145.0278 (100), 133.0285 (34), 117 (14), 105.0343 (29), 89.0377 (21), 77.0378 (14); ^{1}H NMR ($CDCl_3$, 600 MHz) δ 8.13 (1H, s, H-5), 7.66 (1H, d, *J*=9.6 Hz, H-4), 6.99 (1H, s, H-8), 6.39 (1H, d, *J*=9.6 Hz, H-3), 3.82 (3H, s, H-12), 2.84 (1H, hept, *J*=7.0 Hz, H-14), 1.30 (6H, d, *J*=7.0 Hz, H-15, 16); ^{13}C NMR ($CDCl_3$, 151 MHz) δ 175.2 (C-13), 163.9 (C-11), 159.6 (C-2), 157.1 (C-9), 153.5 (C-7), 142.6 (C-4), 132.0 (C-5), 120.5 (C-6), 117.1 (C-3), 116.7 (C-10), 112.7 (C-8), 52.6 (C-12), 34.3 (C-14), 18.8 (C-15, 16); in agreement with published data (Tesso et al., 2005).

8-Methoxypeucedanin (**5**): $C_{16}H_{16}O_5$ MW 288.0993; UV (methanol, λ_{max}, nm): 222, 237 sh, 258, 286 sh, 303; ESI-MS: *m/z* 289.1066 $[M+H]^+$ (calcd for $C_{16}H_{17}O_5$ 289.1071, Δ = 1.56 ppm); MS/MS (40 eV) *m/z* (rel. int.): 274.0816 (10), 259.0587 (100), 244.0347 (97), 219.0276 (42), 216.0423 (26), 204.0032 (13), 176.0110 (22), 148.0163 (7); ^{1}H NMR ($CDCl_3$, 600 MHz) δ 7.69 (1H, d, *J*=9.6 Hz, H-4), 7.18 (1H, s, H-5), 6.30 (1H, d, *J*=9.6 Hz, H-3), 4.21 (3H, s, H-17), 3.87 (3H, s, H-13), 3.20 (1H, hept, *J*=7.0 Hz, H-14), 1.31 (6H, d, *J*=7.0 Hz, H-15, 16); ^{13}C NMR ($CDCl_3$, 151 MHz) δ 160.7 (C-2), 152.7 (C-12), 145.0 (C-7), 144.5 (C-4), 142.9 (C-9), 136.7 (C-11), 132.8 (C-8), 123.1 (C-6), 116.0 (C-10), 114.8 (C-3), 109.7 (C-5), 61.9 (C-13), 61.4 (C-17), 26.3 (C-14), 20.9 (C-15, 16); in agreement with published data (Chinou et al., 2007).

Peucedanin (**6**): $C_{15}H_{14}O_4$ MW 258.0877; UV (methanol, λ_{max}, nm): 223, 231 sh, 256, 283 sh, 298; ESI-MS: *m/z* 259.0950 $[M+H]^+$ (calcd for $C_{15}H_{15}O_4$ 259.0941, Δ = -3.56 ppm); MS/MS (40 eV) *m/z* (rel. int.): 229.0487 (100),

189.0.174 (10), 185.0598 (21), 145.0284 (38), 128.0635 (14), 117.0711 (26); ^{1}H NMR ($CDCl_3$, 600 MHz) δ 7.73 (1H, d, *J*=9.5 Hz, H-4), 7.51 (1H, s, H-5), 7.28 (1H, s, H-8), 6.31 (1H, d, *J*=9.5 Hz, H-3), 3.88 (3H, s, H-13), 3.19 (1H, hept, *J*=7.0 Hz, H-14), 1.29 (6H, d, *J*=7.0 Hz, H-15, 16); ^{13}C NMR ($CDCl_3$, 151 MHz) δ 161.3 (C-2), 153.8 (C-7), 152.9 (C-12), 151.8 (C-9), 144.2 (C-4), 136.5 (C-11), 121.9 (C-6), 116.7 (C-5), 114.9 (C-10), 114.6 (C-3), 100.2 (C-8), 61.9 (C-13), 26.2 (C-14), 20.9 (C-15, 16); in agreement with published data (Shults et al., 2003).

References

Chinou, I.; Widelski, J.; Fokialakis, N.; Magiatis, P.; Glowniak, K. Coumarins from *Peucedanum luxurians*. *Fitoterapia* **2007**, *78*, 448-449.

Edwards, D.J.; Bellevue III; F.H., Woster; P.M. Identification of 6',7'-dihydroxybergamottin, a cytochrome P450 inhibitor, in grapefruit juice. *Drug Metab. Dispos.* **1996**, *24*, 1287-1290.

Shults, E.E.; Petrova, T.N.; Shakirov, M.M.; Chernyak, E.I.; Pokrovskiy, L.M.; Nekhoroshev, S.A.; Tolstikov, G.A.Coumarin compounds from roots of *Peucedanum* (*Peucedanum morisonii* Bess.). *Chem. Sustainable Dev.* **2003**, *11*, 649-654.

Tatum, J.H.; Berry, B.E. Coumarins and psoralens in grapefruit peel oil. *Phytochemistry* **1979**, *18*, 500-502.

Tesso, H.; König, W.A.; Kubeczka, K.H.; Bartnik, M.; Glowniak, K. Secondary metabolites of *Peucedanum tauricum* fruits. *Phytochemistry* **2005**, *66*, 707-713.
